# From Diagnosis to Durability: A Review of the European Bulevirtide Experience and Practical Learnings for CHD Management

**DOI:** 10.1111/liv.70757

**Published:** 2026-06-25

**Authors:** Monica Radu, Margarita Papatheodoridi, Habiba Kamal Khodir, Sabela Lens, Elisabetta Degasperi, Speranta Iacob, Liliana Gheorghe, Katja Steppich, Florin Alexandru Caruntu, Pietro Lampertico, Soo Aleman, Heiner Wedemeyer

**Affiliations:** ^1^ National Institute for Infectious Diseases “Prof. Dr. Matei Bals” Bucharest Romania; ^2^ National and Kapodistrian University of Athens Athens Greece; ^3^ Institute for Liver and Digestive Health, University College London London UK; ^4^ Department of Medicine Huddinge Karolinska Institutet Stockholm Sweden; ^5^ Department of Infectious Diseases Karolinska University Hospital Stockholm Sweden; ^6^ Liver Unit Hospital Clinic, University of Barcelona, FCRB‐IDIBAPS Barcelona Spain; ^7^ Biomedical Research Network in Hepatic and Digestive Diseases (CIBERehd) Madrid Spain; ^8^ Division of Gastroenterology and Hepatology Fondazione IRCCS Ca' Granda Ospedale Maggiore Policlinico Milan Italy; ^9^ Carol Davila University of Medicine and Pharmacy Bucharest Romania; ^10^ Fundeni Clinical Institute Bucharest Romania; ^11^ HepNet Study‐House, German Liver Foundation Medizinische Hochschule Hannover (MHH) Hannover Germany; ^12^ Department of Gastroenterology, Hepatology, Infectious Diseases and Endocrinology Hannover Medical School Hannover Germany

**Keywords:** antiviral agents, bulevirtide, chronic hepatitis D, disease management, hepatitis delta virus

## Abstract

Hepatitis delta virus (HDV) infection causes chronic hepatitis D (CHD), the most severe form of chronic viral hepatitis with a high risk of progression to advanced liver disease. Off‐label pegylated interferon alpha was the primary treatment option in Europe prior to the introduction of bulevirtide, a first‐in‐class HDV entry inhibitor. This review aimed to describe practical learnings around the management of CHD and strategies for supporting patients since bulevirtide 2 mg became the first approved treatment option in Europe. Since bulevirtide's approval, there has been an increased urgency to connect patients to care and real‐world evidence has accumulated, underscoring the importance of reflex testing, increased awareness of HDV among doctors, and harmonization of universal screening guidelines in Europe to facilitate care for patients early in the disease course. Discussions with patients have shifted from preparing for disease progression to living with a long‐term, daily treatment regimen, and clinical experience provides insight into best practices for navigating these conversations; it is critical for doctors to set evidence‐based treatment expectations and provide ongoing guidance and support to patients. Real‐world evidence demonstrates high treatment adherence and persistence; these studies and clinical trial data also indicate that long‐term bulevirtide monotherapy is safe and effective, even in patients with compensated cirrhosis, may improve liver fibrosis measurements, and potentially reduce the risk of liver‐related events and decompensation, which are important motivators for patients. Future research is needed to establish the safety and effectiveness of bulevirtide in certain special populations and determine the optimal treatment duration.

## Key Points

- Clinical practice and perspectives surrounding chronic hepatitis D (CHD) management have evolved since the approval of bulevirtide monotherapy in Europe.
- With bulevirtide's approval, there has been an increased urgency to connect patients with CHD to care, reflected in increased screening efforts and the accumulation of real‐world evidence.
- Universal screening with reflex testing is critical to facilitate timely patient identification and linkage‐to‐care.
- Real‐world evidence supports the long‐term safety and effectiveness of bulevirtide monotherapy across diverse patient populations and demonstrates high treatment adherence and persistence.
- It is critical for physicians to set evidence‐based treatment expectations and provide patient‐centred guidance and support.

**Abbreviations**
AE: adverse event
ALT: alanine aminotransferase
BLV: bulevirtide
CHD: chronic hepatitis D
DNA: deoxyribonucleic acid
EASL: European Association for the Study of the Liver
EMA: European Medicines Agency
HBsAg: hepatitis B surface antigen
HBV: hepatitis B virus
HCC: hepatocellular carcinoma
HCP: healthcare professional
HCV: hepatitis C virus
HDV: hepatitis delta virus
HIV: human immunodeficiency virus
HRQoL: health‐related quality of life
IFN: interferon
LOD: limit of detection
LRE: liver‐related event
LSM: liver stiffness measurement
MoA: mechanism of action
NA: nucleos(t)ide analog
NTCP: sodium taurocholate co‐transporting polypeptide
Peg‐IFNα: pegylated interferon alpha
PRO: patient‐reported outcome
RNA: ribonucleic acid
TE: transient elastography

## 1 Introduction

Hepatitis delta virus (HDV) infection causes chronic hepatitis D (CHD), the most severe form of chronic viral hepatitis, which is associated with a high risk of progression to compensated cirrhosis, decompensated cirrhosis, hepatocellular carcinoma (HCC), and liver‐related mortality [1, 2]. HDV is a defective virus requiring the presence of hepatitis B virus (HBV), through either a super‐infection or acute co‐infection that may develop into CHD, relying on hepatitis B surface antigen (HBsAg) and host cell machinery for replication and propagation [2].

HDV prevalence among patients with HBV is estimated at 3% in Europe and 5% globally (though some epidemiological studies have cited up to 15% [3, 4]), or approximately 12 million patients with HDV infection worldwide [5]. HDV prevalence estimates vary across studies, and the true prevalence remains difficult to determine due to geographic heterogeneity, limited systematic population‐based surveys, variability in screening populations (e.g., general vs. hospital‐based populations), inconsistent screening strategies, suboptimal awareness of HDV, and a lack of standardized diagnostic protocols [5, 6].

Treatment options for CHD were historically limited. Until recently, off‐label pegylated interferon alpha (Peg‐IFNα) monotherapy was the only available treatment for CHD, although many patients are ineligible for Peg‐IFNα due to contraindications (including decompensated cirrhosis and extra‐hepatic contraindications) and its use is associated with substantial side effects, which may lead to treatment discontinuation in some patients [7, 8, 9]. Bulevirtide (BLV) 2 mg received conditional approval from the European Medicines Agency (EMA) in 2020 and full approval in 2023, and is the first and only approved treatment option for CHD in Europe and certain countries outside Europe [10, 11, 12].

With the introduction of BLV, clinical practice for CHD management and the patient journey have evolved substantially. The aim of this review is to integrate findings from published literature and expert clinical perspectives to describe practical learnings for CHD management in Europe since the approval of BLV 2 mg, spanning patient identification, treatment initiation, and long‐term treatment management.

## 2 Patient Finding and Diagnosis

HDV prevalence and epidemiology remain heterogeneous across Europe [6], and have been impacted by migration from endemic areas [13]. Published data and real‐world clinical perspectives indicate that the majority of patients with CHD in Europe are migrants from high‐prevalence regions [13, 14]. In clinical practice, patient identification and linkage‐to‐care within vulnerable populations (e.g., migrant, rural, and socioeconomically disadvantaged patients) remains challenging, as these patients may not attend conventional healthcare circuits, face socioeconomic or language barriers to accessing care, or are lost‐to‐follow‐up after initial contact [14]. The heterogeneity in HDV prevalence across Europe directly impacts policy decisions, health equity, and real‐world treatment strategies, and it is critical to consider factors that impact CHD risk to facilitate timely diagnosis. However, the approval of BLV as a therapeutic option for CHD has led to renewed interest in expanding efforts to facilitate patient finding and treatment initiation in many European countries.

### 2.1 Screening and Diagnosis

The European Association for the Study of the Liver (EASL) HDV and HBV guidelines recommend screening all HBsAg‐positive patients for HDV antibodies (anti‐HDV; with retesting as clinically indicated) and testing for HDV ribonucleic acid (RNA) in all anti‐HDV‐positive patients to confirm active HDV infection [15, 16]. However, universal HDV testing has not been consistently applied in clinical practice, with screening rates of ≤ 26% of HBsAg‐positive patients in some real‐world studies, and particularly low rates in certain subpopulations such as pregnant women [17, 18, 19], emphasizing the importance of widespread screening and coordination across care teams. BLV's availability has been critical in building support for programs that facilitate universal screening; with an approved treatment option, there is an increased urgency for patient finding and timely treatment initiation.

While some specialized clinics have systematically screened all HBsAg‐positive patients for decades, upstream delays in patient identification and linkage‐to‐care may originate from primary or non‐tertiary care settings where HDV awareness and routine testing is less‐established. For example, in Spain, higher HDV screening rates were observed in specialized versus primary care settings [20]. Delayed anti‐HDV screening has been associated with a higher risk of liver‐related complications [21], and 33% of patients with CHD in a multicenter study had cirrhosis at the time of HDV diagnosis [22]. Prospectively recalling HBsAg‐positive patients who were not screened for HDV may improve patient finding [23], and it is important to encourage early screening and referral, particularly in non‐specialized settings, to help mitigate the burden of CHD. In some clinical practices, the high proportion of patients with CHD requiring liver transplantation may reflect delays in HDV diagnosis and specialist referral; in Romania, patients referred to tertiary clinics often already have advanced fibrosis and are seeking specialist care.

Single‐step anti‐HDV reflex testing has been efficient in expanding and simplifying screening efforts across real‐world healthcare settings, and may help facilitate patient finding among populations disproportionately affected by HDV (e.g., migrants) [20, 24, 25, 26, 27]. In the French ANRS CO22 HEPATHER study, 90% of viremic patients with HDV infection were migrants and 62% were living in poverty, a significantly higher proportion than among patients with HBV mono‐infection [28]. Vulnerable, at‐risk patients are often more difficult to diagnose early if anti‐HDV reflex testing is not performed at first contact; returning to the clinic can be challenging for these patients. While performing point‐of‐care reflex testing for HDV RNA (i.e., positive anti‐HDV test followed by ‘reflex’ point‐of‐care HDV RNA testing) would be ideal, such point‐of‐care tests are not yet available. Point‐of‐care nucleic acid tests have been developed for other viruses, e.g., HBV [29], and this remains an area of further development in HDV. Structured strategies are also needed to effectively engage these at‐risk groups in the care cascade, including on‐site language interpreters and clinical collaboration with regional health departments for outreach. Knowledge gaps among healthcare professionals (HCPs) regarding screening recommendations may also contribute to inconsistent anti‐HDV testing practices; while HDV testing was acceptable to United Kingdom HCPs in a 2024 study, only 67% correctly cited EASL HDV guidelines [30]. Targeted educational interventions for both clinicians and patients regarding the HDV care cascade are needed to improve linkage‐to‐care, especially for vulnerable groups.

**FIGURE 1:**
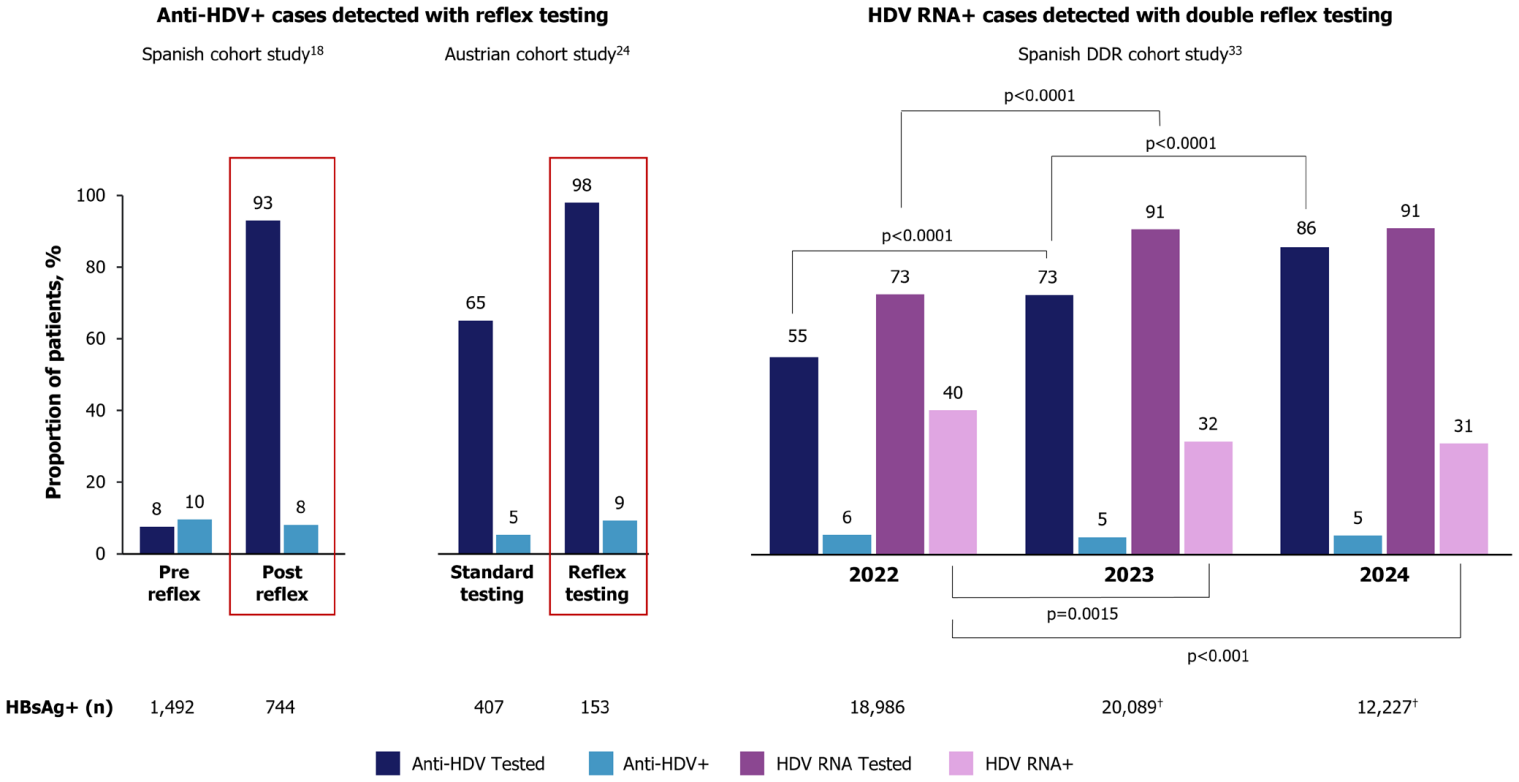
Improvement in HDV diagnosis rates with reflex and double reflex testing among HBsAg‐positive patients [18, 24, 33]. Anti‐HDV, HDV antibodies; DDR, Delta Double Reflex; HBsAg, hepatitis B surface antigen; HDV, hepatitis delta virus; RNA, ribonucleic acid; +, positive. ^†^Among patients without a previous HDV test.

World Health Organization guidance indicates that double reflex testing of anti‐HDV and HDV RNA in HBsAg‐positive patients may help facilitate diagnosis of HDV infection [29]. Double reflex testing has been a successful strategy to address low screening rates and facilitate linkage‐to‐care, particularly in countries with low HBV and high HDV prevalence (Figure 1) [31]. In Romania, reflex testing of all HBsAg‐positive patients is already implemented by protocol [32]. Elsewhere, targeted reflex testing strategies have been successfully implemented prior to BLV's approval, such as in Spain, where a double reflex testing programme increased anti‐HDV testing from 55% in 2022 to 86% in 2024 [33]. Beyond streamlining patient finding, double reflex testing is expected to lead to cost savings and pose little economic burden on healthcare systems [31]. Strategies to increase HDV detection through reflex testing programs, at‐risk population outreach, and HCP education have improved patient identification and linkage‐to‐care, and may help reduce the downstream clinical and economic burden of disease [17, 21, 34].

### 2.2 Linkage‐to‐Care

As individuals with CHD may have already progressed to cirrhosis by the time of diagnosis and may experience worsened liver‐related outcomes when HDV screening is delayed [21, 22], timely patient identification is critical to establish specialist referral and facilitate appropriate disease management, including treatment and long‐term monitoring. While liver biopsy represents the gold standard for liver disease staging, given its invasive and expensive nature, non‐invasive tests are increasingly used to monitor disease progression and treatment response [2, 35]. The global recognition of transient elastography (TE) as a reliable tool for fibrosis staging represents a key advancement in best practices for CHD management, and regular TE monitoring should be integrated into follow‐up care where available [35, 36, 37]. In two large real‐world studies, liver stiffness measurement (LSM) by TE with cutoffs of ≥ 15.2 and ≥ 10.0 kPa have been proposed for the diagnosis of cirrhosis and advanced fibrosis, respectively, while TE values < 6.0 kPa likely exclude significant fibrosis [36, 37]. Baveno VII cutoffs for non‐invasive diagnosis of clinically significant portal hypertension have also been recently validated in CHD [38]. Where TE is technically limited or infeasible (e.g., in patients with morbid obesity or ascites) [39], 2D‐ or point shear wave elastography are the preferred ultrasound‐based alternatives; magnetic resonance elastography, while highly accurate, should generally be reserved for cases where ultrasound‐based elastography methods are not available or technically inadequate.

Even with these advances in fibrosis staging, limited awareness of HDV and its disease course among doctors and patients may contribute to linkage‐to‐care gaps, and targeted strategies to relink patients to care following diagnosis have been successful [40]. In a Spanish database review, 29% of patients with HDV infection were not linked‐to‐care; direct outreach to patients with HDV infection who were eligible for contact and linkage to specialist care led to successful reintegration of 100% of these patients into care [40]. With the availability of an approved treatment option, there has been an increased focus on re‐engaging previously diagnosed but untreated patients, as these individuals may now benefit from linkage to active treatment.

## 3 Treatment Initiation

### 3.1 Treatment Regulations and Guidelines

Per the 2023 EASL HDV guidelines, BLV treatment should be considered for all patients with CHD and compensated liver disease [15], and 2025 EASL HBV guidance suggests patients with decompensated liver disease may also receive BLV depending on their risk–benefit assessment [16]. Patients with advanced fibrosis have been prioritized for treatment in some countries, including Sweden, while other countries, including Italy, Germany, and Romania, do not have such restrictions. Patients with LSM < 10 kPa may still progress to cirrhosis and other liver‐related events (LREs) [41], reinforcing the urgency to initiate BLV in all eligible patients.

### 3.2 Best Practices for BLV Initiation

In our collective experience treating patients with BLV in our clinical practices, delays in initiating BLV are often related to patient concerns about daily subcutaneous injections and the prospect of long‐term treatment, even among patients with severe liver disease. Regular clinical follow‐up visits that allow time for thorough discussion, education, support, and shared decision‐making have helped to make patients feel more comfortable with long‐term therapy and may mitigate delays in initiating BLV.

Clearly explaining the practical implications of BLV's regimen using a patient‐centred approach is crucial. Patients often ask how daily injections fit into their routines, particularly regarding work schedules and optimal time of day for injections, and managing injections when travelling, as well as potential drug–drug interactions and dietary requirements. It is important for doctors to proactively answer these questions in patient conversations: there are few drug–drug interactions with BLV (although co‐medications should be checked against the Summary of Product Characteristics [SmPC]), BLV should be taken at the same time every day (even while travelling), and there are no dietary restrictions [42]. Patients should be counselled on practical aspects of BLV preparation and administration per the SmPC [42]. Preferred injection sites include the abdomen and upper thighs, with regular rotation to minimize injection‐site reactions. If a dose is missed, it should be administered as soon as possible if < 4 h have elapsed; otherwise, it should be skipped and the next dose taken at the usual scheduled time. Patients should inform their HCP of missed or duplicated doses. BLV vials should be refrigerated at 2°C–8°C and protected from light, and the reconstituted solution used immediately. Chemical and physical in‐use stability has been demonstrated for 2 h at room temperature (≤ 25°C). During travel, vials may be transported in a cooled container and refrigerated upon arrival, maintaining appropriate sharps disposal.

We have found that offering examples of other conditions with long‐term, daily treatment regimens, such as insulin injections for diabetic patients, may resonate with patients, and leveraging individualized planning and support from clinic staff (e.g., nurses) is often helpful. Structured, in‐person education supported by visual aids are also successful in helping patients, including those with limited literacy, learn correct injection techniques and maintain adherence.

Our collective clinical experience suggests that patients' expectations are often based on those of the treating physician. It is therefore important to clearly and objectively explain treatment goals, and doctors may share clinical trial and real‐world findings with patients to help set realistic treatment expectations, emphasizing the key aims of halting disease progression, reducing the risk of liver‐related complications (e.g., cirrhosis, HCC), and potential regression of advanced fibrosis. Experienced clinicians have reported that most patients, especially younger patients, are motivated to initiate and adhere to BLV when they clearly understand these goals. Additionally, patients (especially Peg‐IFNα‐experienced) often ask about side effects and quality of life; it is important to highlight the optimal tolerability profile of BLV early in conversations with these patients to reassure them, while also being clear about potential side effects (e.g., injection‐site reactions). New doctors should discuss cofactors (e.g., diabetes, obesity, alcohol use) that may increase risks of LREs [15], as managing these factors is critical to improving long‐term outcomes.

Ultimately, clear, comprehensive information and ongoing support can help patients feel more comfortable with BLV initiation and long‐term management.

## 4 Current Treatment Landscape

While nucleos(t)ide analogs (NAs) for HBV treatment do not substantially impact CHD progression [43, 44], HBV infection should be managed as clinically indicated in patients with compensated or decompensated cirrhosis and all patients with HBV deoxyribonucleic acid (DNA) levels ≥ 2000 IU/mL [15, 16].

Prior to the approval of BLV 2 mg in Europe, off‐label Peg‐IFNα regimens were the mainstay of managing HDV infection and continue to be used in some countries [7]. Peg‐IFNα monotherapy leads to sustained off‐treatment inhibition of HDV replication in ~20% of patients, is associated with adverse events (AEs; including hematologic side effects and some health‐related quality of life [HRQoL] impairments), and is contraindicated in many patients (e.g., decompensated cirrhosis) [7, 8, 45, 46].

BLV is a first‐in‐class antiviral therapy that acts by inhibiting the sodium taurocholate co‐transporting polypeptide (NTCP), a bile acid transporter that is also a receptor for the entry of HBV and HDV into hepatocytes [42]. BLV's approval represents a substantial change in therapeutic options for HDV infection, particularly among patients reluctant to start Peg‐IFNα treatment, who experienced side effects with Peg‐IFNα, or were Peg‐IFNα non‐responders. In discussions with patients, explaining the advances in short‐ and long‐term efficacy and tolerability with BLV treatment compared to Peg‐IFNα can help to appropriately frame treatment expectations.

### 4.1 On‐Treatment BLV Effectiveness & Clinical Benefit of BLV

Serum HDV RNA and alanine aminotransferase (ALT) are key surrogate markers of HDV infection. ALT is released into the peripheral circulation as a consequence of hepatocellular injury associated with HDV infection, whereas serum HDV RNA reflects the level of circulating virions and ongoing viral replication within infected hepatocytes [47, 48]. Accordingly, established surrogate endpoints for assessing treatment efficacy include virologic response (achievement of undetectable HDV RNA or a ≥ 2 log_10_ IU/mL decline in HDV RNA levels); biochemical response (ALT normalization); combined virologic and biochemical response; and sustained undetectable HDV RNA [15, 49].

**FIGURE 2:**
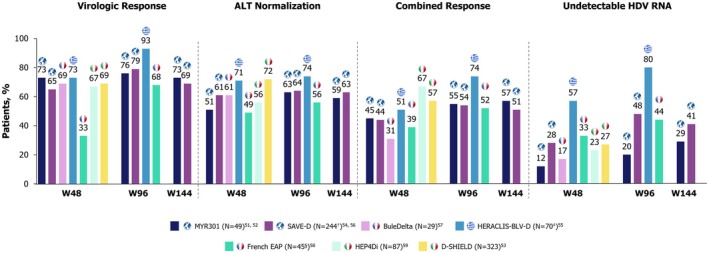
Summary of BLV 2 mg monotherapy effectiveness across MYR301 and real‐world cohort studies [51, 52, 53, 54, 55, 56, 57, 58, 59] ALT, alanine aminotransferase; BLV, bulevirtide; EAP, early access program; HDV, hepatitis delta virus; RNA, ribonucleic acid; W, week. ^†^ *n* = 244 at W48 and W96, *n* = 78 at W144; this timepoint is a sub‐analysis among patients reaching W144; ^‡^ *n* = 70 at 12 months (representing W48) and *n* = 46 at 24 months (representing W96); ^§^ *n* = 43 (virologic response and undetectable HDV RNA), *n* = 44 (combined response), or *n* = 45 (ALT normalization) at 12 months (representing W48); *n* = 25 (virologic response, combined response, undetectable HDV RNA) or *n* = 27 (ALT normalization) at 24 months (representing W96).

The favourable efficacy profile of BLV monotherapy in patients with and without compensated cirrhosis has been established in the MYR301 Phase 3 clinical trial [50, 51]; by 144 weeks of treatment, 73%, 59%, and 29% of patients achieved virologic response, biochemical response, and undetectable HDV RNA, respectively [52]. Real‐world evidence across European cohorts also demonstrates the effectiveness of BLV monotherapy in achieving disease control among patients with and without cirrhosis, highlighting the key role of BLV even in populations who are often difficult‐to‐treat (Figure 2) [53, 54, 55]. Together, clinical trial data and real‐world experience provide important guidance for treatment decisions and patient counselling.

Setting realistic expectations on short‐ and long‐term response to BLV treatment is crucial. Patients may have high expectations for treatment outcomes, especially regarding reductions in viremia; however, the timing of decreases in viremia with BLV is not comparable to that caused by a direct antiviral agent (e.g., NAs for HBV) due to their distinct mechanisms of action (MoAs) [51, 60]. As BLV binds the NTCP receptor, *de novo* HDV infection of hepatocytes is blocked, leading to a rapid decline in ALT [51, 60]. Meanwhile, peripheral HDV RNA levels decline more gradually as pre‐existing HDV‐infected cells die out due to natural turnover, leading to biopsy‐proven reductions in the number of infected hepatocytes [60]. It is important to proactively clarify to patients that achievement of virologic response is expected to occur more gradually compared to biochemical response (which can improve within the first 6–12 months of treatment), but that HDV RNA decreases with BLV represent actual reductions in the liver burden [60]. Simplifying and explaining BLV's MoA can offer helpful context for these dynamics; for patients with limited health literacy, providers may focus on the key practical message that gradual HDV RNA decline is expected and reflective of BLV's MoA.

These dynamics are reflected in the results of MYR301, which demonstrated improvements in virologic and biochemical response during 144 weeks of BLV monotherapy [52]. In a MYR301 sub‐analysis, 82% of partial virologic responders and 43% of non‐responders at Week 24 achieved virologic response at Week 96 [51]. Additionally, a MYR202/MYR301 resistance analysis in virologic non‐responders did not show reduced virological sensitivity to BLV, highlighting its high barrier to resistance [61]. These findings are reflective of BLV's MoA and gradual impact on HDV RNA, supporting the value of continuing BLV treatment even in patients without initial response (i.e., response within 6 months of treatment initiation).

BLV treatment through MYR301 Week 96 also led to ALT improvements irrespective of virologic response [51], highlighting the importance of continuing treatment even in cases of partial or no virologic response. In line with clinical trials, a third of suboptimal virologic responders in the real‐world SAVE‐D study achieved a > 50% decrease in ALT levels (vs. baseline) with BLV, and in D‐SHIELD, biochemical response and ALT reductions were observed in 35% and 51%, respectively, of virological non‐responders at Week 72 of treatment [53, 54]. In a German cohort study, a significant decline in ALT was reported as early as 12 weeks on‐treatment in virologic responders and non‐responders [62]. Together, these results suggest that ALT normalization is achievable early and irrespective of virologic response, bearing particular importance in patients with advanced fibrosis who are prioritized for treatment in certain countries.

BLV may also facilitate a reduced risk of LREs and improvements in disease progression, although it is important to emphasize to patients that these potential treatment benefits can take time. A case–control analysis of patients with HDV‐related cirrhosis suggested that, compared to no treatment, a 2‐year course of BLV monotherapy resulted in a reduced risk of decompensation, but not of HCC [63]; this was not an unexpected finding in patients with such advanced liver disease. SAVE‐D observed a low 3‐year cumulative incidence of *de novo* decompensation (3%) and HCC (5%) in patients with HDV‐related cirrhosis receiving 192 weeks of BLV monotherapy [64]. Improvements in LSM have been observed over 144 weeks of BLV monotherapy in MYR301 [52], and in SAVE‐D, significant reductions in LSM values were observed between baseline and Week 96 of BLV treatment independently of treatment response [54]. Real‐world studies have also found improvements in other serological markers of liver fibrosis (e.g., Fibrosis‐4 index and aspartate aminotransferase to platelet ratio scores) with BLV [54, 62]. Potential regression of liver fibrosis may be discussed as a long‐term possibility for patients; however, patients should be advised that meaningful structural liver improvement may take years rather than months.

In clinical practice, clear communication with patients about objective signs of treatment response (e.g., improvements in biochemical markers and reductions in viral load) and the clinical implications of these changes over time (e.g., how these responses relate to long‐term liver health) can help to reinforce patient motivation and engagement with both therapeutic regimen and follow‐up care.

### 4.2 Predictors of On‐Treatment BLV Response

Given the natural history and substantial burden of CHD, it is critical to evaluate meaningful predictors of HDV RNA reduction or undetectability and long‐term clinical outcomes. In MYR301, low baseline HDV RNA and HBsAg levels were predictors of undetectable HDV RNA at the end of BLV treatment [65]. In SAVE‐D, lower baseline HDV RNA was associated with achieving HDV RNA undetectability at 48 weeks of BLV treatment, while higher baseline HDV RNA and HBsAg were associated with virologic response [54]. While low baseline viremia has been identified as a consistent predictor of BLV response, further research is needed to determine if other robust and clinically meaningful predictors exist. However, the lack of consistent predictors of response alongside the high rates of response to BLV monotherapy observed in clinical trials and real‐world studies highlights the effectiveness and role of BLV in CHD management across diverse patient populations.

### 4.3 On‐Treatment BLV Safety

Patient questions related to BLV's side effects are common in clinical practice, especially among patients with Peg‐IFNα experience. Explaining the BLV's safety profile in clinical and real‐world populations, including subpopulations with compensated cirrhosis, and emphasizing the absence of systemic side effects may help reassure patients and reduce barriers to treatment initiation.

In MYR301, BLV was well‐tolerated through 144 weeks; the most common AEs were mild, serious AEs were uncommon and unrelated to BLV, and discontinuation due to treatment‐related AEs was not observed [50, 51, 52]. Similarly, BLV was safe and well‐tolerated even in patients with compensated cirrhosis through 96 weeks of treatment in SAVE‐D, with low rates of pruritus (10%), *de novo* LREs (5%), and injection site reactions (3%), and only 5% discontinued treatment [54]. The positive safety profile of BLV across patients with and without cirrhosis is also supported by the results of other real‐world studies, including D‐SHIELD and HERACLIS‐BLV‐D [53, 55].

Increased serum bile acid levels during BLV treatment were observed in MYR301 and real‐world studies (e.g., SAVE‐D); however, these elevations were expected due to BLV's inhibition of NTCP, a bile acid transporter, and were asymptomatic with no correlation to clinical sequelae [50, 51, 54, 66]. In our combined clinical experience, we have not observed clinically relevant issues related to bile acid elevations on BLV, and these elevations are not considered to be a major safety concern.

### 4.4 Patient‐Reported Outcomes

CHD is associated with substantial negative HRQoL impacts, including psychological and physical well‐being impairments, and patients with HDV infection may experience worse patient‐reported outcomes (PROs) than patients with HBV mono‐infection [67]. MYR301 demonstrated significant improvements in patient‐reported physical and disease‐specific HRQoL domains through 48 weeks of BLV monotherapy compared to controls, indicating symptom and functional benefits associated with BLV [68]. In a multicenter European cohort study, patients receiving BLV monotherapy for ≥ 6 months reported better disease‐specific HRQoL than treatment‐naïve patients, despite more severe disease at the survey date [69], and similar HRQoL improvements with BLV were reported among German patients in vitality, mental health, and pain domains [70].

In a recent survey, patients with CHD reported seeking accessible, comprehensive information on HDV, treatment options, and mental and physical health resources [71]. This can be achieved through direct patient support; for example, a pharmacist‐led intervention in Italy consisting of medication counselling, proactive surveillance, and individualized support for patients with CHD receiving BLV contributed to significant HRQoL improvements [72]. In practice, the psychological burden of CHD should be discussed with patients [73], and consideration of PROs can facilitate holistic disease management aligning with patient needs.

### 4.5 Long‐Term Management: Patient Engagement and Monitoring

**FIGURE 3:**
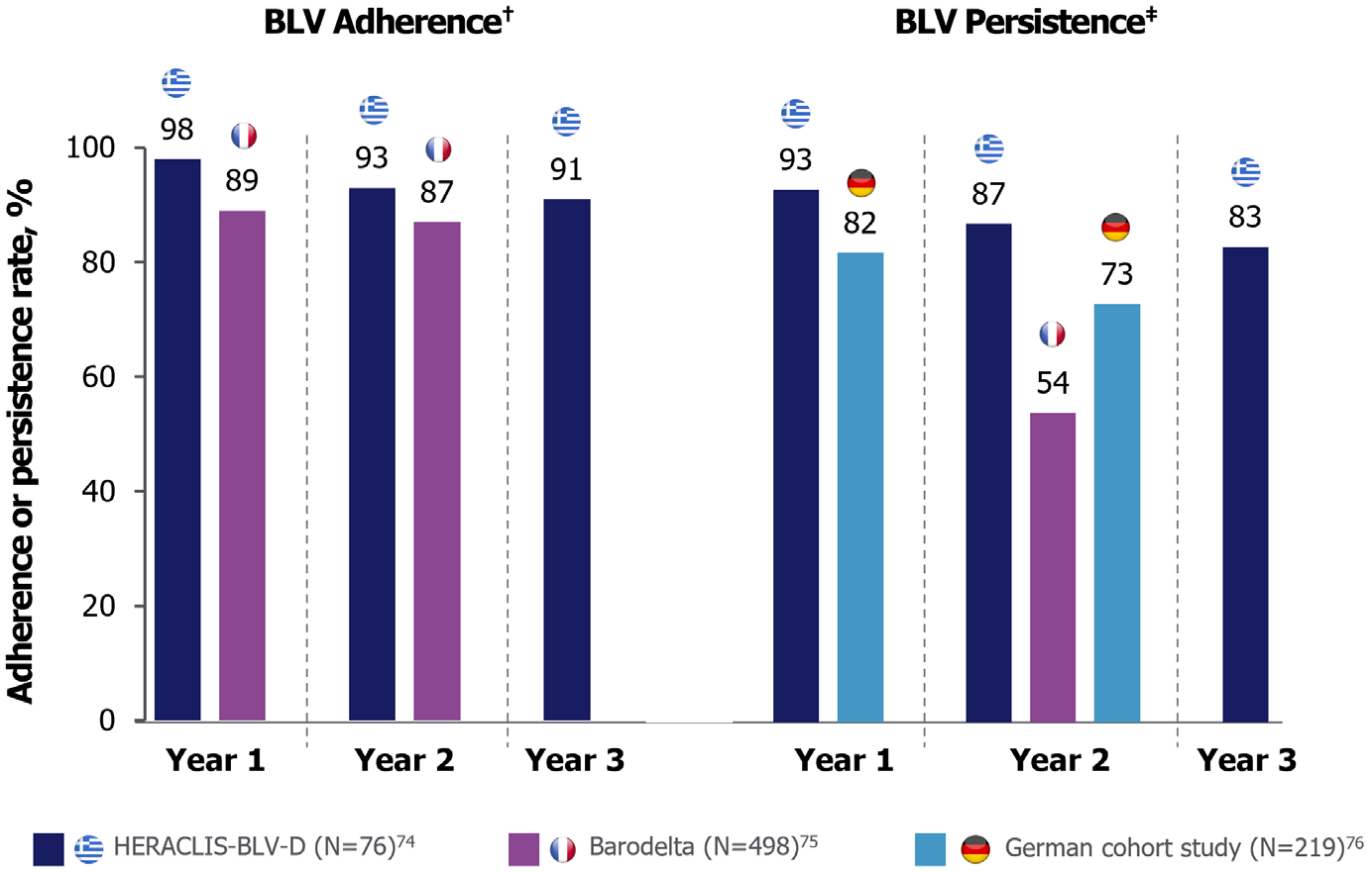
Summary of treatment adherence and persistence to BLV monotherapy in real‐world studies [74, 75, 76]. BLV, bulevirtide. ^**†**^Determined by the rates of monthly prescriptions of BLV boxes of 30 vials (percentage of executed prescriptions over certain months divided by the number of months) [74] or estimated by the Medication Possession Ratio [75]; ^**‡**^Defined as the probability of not having discontinued treatment, estimated using the Kaplan–Meier method in Asselah et al. [75] and Nierhoff et al. [76]

Since BLV's approval, the focus of conversation with patients has shifted from preparing patients for disease progression to discussing treatment goals and managing a long‐term, daily treatment regimen. Despite BLV's daily regimen, treatment adherence and persistence remain high in clinical trials (≥ 93% adherence through Week 144 in MYR301) and real‐world studies across Europe (Figure 3) [52, 74, 75, 76].

Technical aspects of BLV administration as a daily subcutaneous injection were not a barrier to adherence in a recent survey [78]. Additionally, in a German study reporting high overall patient satisfaction with the practical aspects and tolerability of BLV, while language proficiency was associated with patient satisfaction, it did not impact practical difficulties with BLV administration [79]. Adherence may be impacted by challenges with integrating treatment into daily life; lack of robust, continuing education on BLV's treatment course (e.g., side effects, managing missed doses); and structural inequalities in BLV availability across geographic regions [78]. Obstacles to treatment compliance were more often reported by migrants and younger patients, highlighting potential systemic barriers to care [78].

**FIGURE 4:**
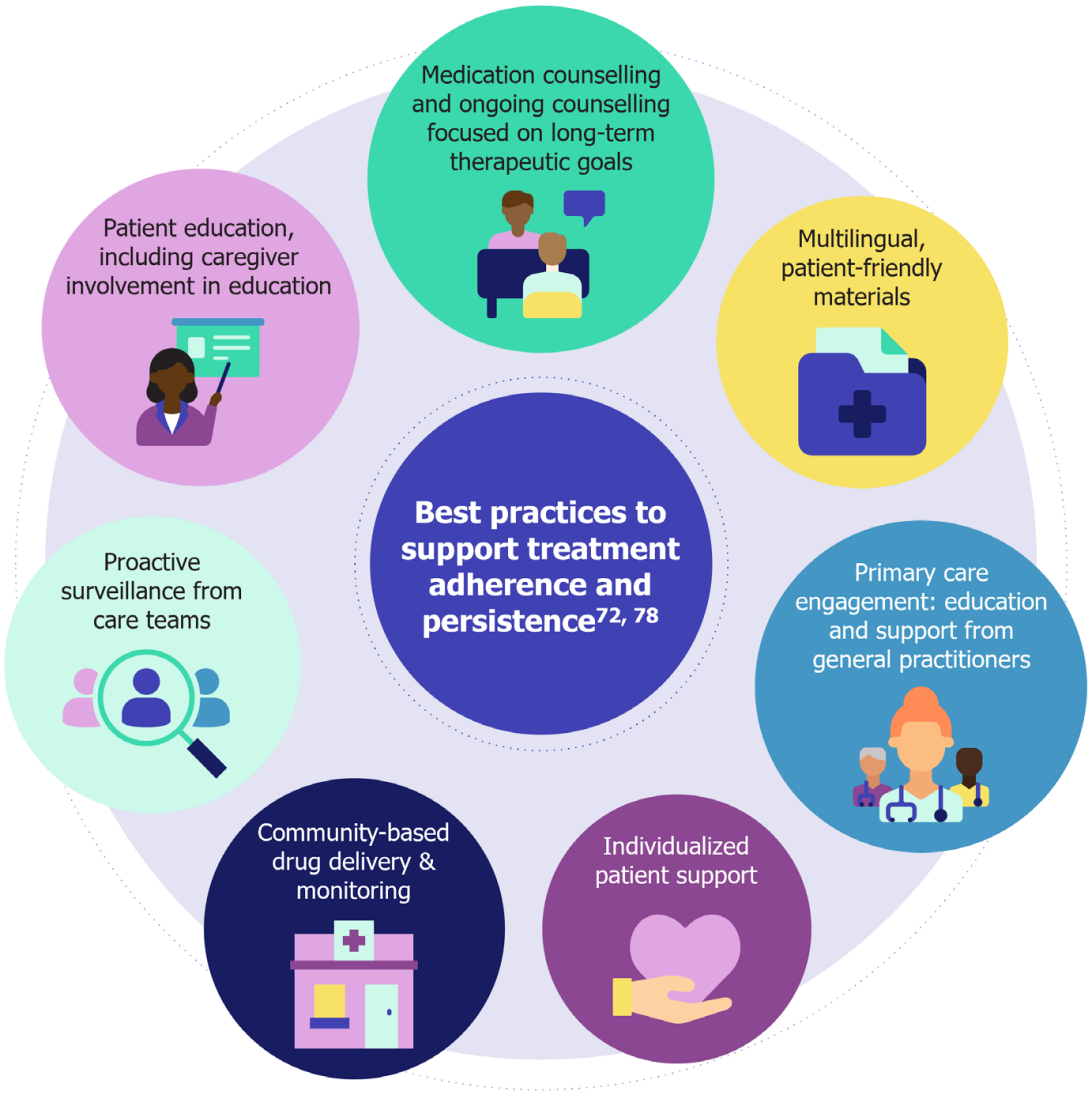
Best practices to support BLV treatment adherence and persistence [72, 78].

Continuity of care via regular follow‐up visits, clear patient education, and patient‐centred, multidisciplinary support strategies tailored to local care structures can promote BLV adherence, including through involvement of general practitioners, specialized nurses, pharmacists, and other trained HCPs (Figure 4).

During treatment and following treatment discontinuation, EASL guidelines recommend monitoring disease parameters (including virologic markers, biochemical markers, and LREs) [15], and it is important to clearly communicate expectations with patients throughout monitoring visits. Visualizing longitudinal trends in an individual's treatment response, either by manually compiling biologic, virologic, and imaging results across clinic visits or via registry tools where available, can support the wider care team and strengthen patient confidence in ongoing treatment.

Regular HDV RNA viral load testing should be conducted in line with EASL practice guidelines [15], and as assays have been found to differ in their sensitivity to HDV RNA, where assays used in the real world (e.g., EurobioPlex, limit of detection [LOD] < 10 IU/mL) may not be as sensitive as those used in clinical trials (e.g., Robogene HDV RNA Quantification Kit 2.0, LOD 6 IU/mL) [80, 81], clinicians should use the most sensitive assay available and use the same assay during and following treatment to ensure an accurate understanding of changes in a patient's HDV RNA levels over time. Clinicians should be aware of assay limitations and sensitivity variation when interpreting HDV RNA results. While the assays used in clinical trials may not always be available in real‐world settings, BLV's approval has facilitated commercial development and availability of sensitive assays, and further studies evaluating sensitivity of assays with different performance characteristics and the establishment of appropriate cutoffs are needed. The development of digital droplet PCRs may overcome limitations of previous HDV RNA assays and ensure more robust quantifications with very high sensitivity [82]. As previously discussed, TE is an important tool for fibrosis staging and monitoring disease progression in patients with CHD [36, 37]. Regular disease monitoring is also critical to identify and facilitate treatment for ALT flares, particularly in the event of treatment discontinuation or interruption [15]; based on our clinical experience, ALT flares may be a concern in patients with cirrhosis and may lead to severe hepatic failure if not appropriately monitored and managed with potential rescue therapy via BLV re‐treatment.

### 4.6 Clinical Management in Special Populations

Real‐world experience and considerations for treating special populations have accumulated since BLV's approval (Table 1). It is important to carefully evaluate, discuss, and tailor goals of therapy for these patients, who should also be managed with optimized HBV suppression and other treatment strategies (e.g., standard care for cirrhosis, HCC, or liver transplantation) where relevant.

**TABLE 1:** Select real‐world studies of BLV 2 mg monotherapy and clinical management implications in special populations.

Population	Real‐world studies	Study design	BLV effectiveness	BLV safety	Additional outcomes
Patients with decompensated cirrhosis	Dietz‐Fricke et al., 2024 [83]	Cohort study across German, Austrian, and Italian centres of patients with CP B cirrhosis (*n* = 19) treated with BLV monotherapy; median observation of 41 weeks	Virologic response: 74%; biochemical response: 74%; combined response: 42%	No AEs were found to be related to BLV treatment	47% improved from CP B to A
	Dietz‐Fricke et al., 2023 [62]	German cohort study (*n* = 114) including 4 CP B patients and 1 CP C patients treated with BLV monotherapy; mean observation period of 38 weeks	All CP B and C patients achieved virologic response; all but one showed decreasing ALT levels and rising platelet counts	No drug‐related AEs leading to BLV discontinuation	—
	Papatheodoridi et al., 2025 [55]	Greek HERACLIS‐BLV‐D study (*n* = 76) including 7 patients with baseline decompensated cirrhosis treated with BLV for 2 years	Among patients with decompensated cirrhosis at baseline (*n* = 7), 4 achieved HDV RNA undetectability, 1 achieved virological response, and 1 did not achieve virologic response by 1 year of treatment; 1 patient without virologic response died due to complications of decompensated cirrhosis	No drug‐related SAEs; no discontinuations due to AEs	Patients with decompensated cirrhosis experienced reversion of liver decompensation by 1 year of BLV monotherapy
	Meszaros et al., 2025 [84]	French cohort study in patients with HDV on the liver transplantation waiting list receiving BLV (*n* = 20) for at least 48 weeks; 7 patients had decompensated cirrhosis at baseline	Among patients with decompensated cirrhosis at baseline (*n* = 7), 43% achieved virologic response and 71% achieved biochemical response	AEs were generally mild and manageable	60% of patients with CP C improved to CP A with BLV; 43% of patients with decompensated cirrhosis experienced liver function improvement while on BLV and were subsequently removed from the transplantation waiting list
Patients with HCC	Meszaros et al., 2025 [84]	French cohort study in patients with HDV on the liver transplantation waiting list receiving BLV (*n* = 20) for at least 48 weeks; 8 patients had active HCC at baseline	—	AEs were generally mild and manageable	BLV treatment led to HCC downstaging in 1 patient and 5 patients with HCC received loco‐regional therapy due to their improved biochemical response, ultimately reducing liver transplantation waitlist drop‐out rates
Patients receiving transplantations	Pinchera et al., 2024 [85]	Case study of a patient with HDV who received 6 months of BLV treatment following kidney transplantation	Achieved virologic response, biochemical response, and undetectable HDV RNA within 2 months of BLV treatment	BLV was generally well‐tolerated	—
Patients with HIV and HDV co‐infection	Degasperi et al., 2025 [54]	Multicenter SAVE‐D study (*n* = 244) of 96 weeks of BLV monotherapy; included 24 patients with HDV/HIV co‐infection	Patients with HDV/HIV co‐infection had comparable response to those without HIV co‐infection	BLV was generally safe and well‐tolerated	HIV RNA remained undetectable; no significant changes in CD4 count
	de Lédinghen et al., 2024 [86]	French BuleDelta study of patients with HDV/HIV co‐infection (*n* = 38) treated with BLV with or without Peg‐IFN‐alpha for at least 48 weeks	58% of patients achieved virologic response	47% of patients had AEs; 13 patients had SAEs	No impact of treatment on CD4 count or HIV viral load
	Visco Comandini et al., 2023 [87]	Italian cohort study (*n* = 13) assessing BLV monotherapy, including 5 patients with HDV/HIV co‐infection; median treatment duration of 11 months	Comparable combined response rates between patients with HDV/HIV and patients with HDV alone (60% vs. 66%)	BLV was generally well‐tolerated, with no discontinuations related to drug toxicity	HIV RNA remained undetectable; increase in CD4 and CD8 cells during treatment

Abbreviations: AE, adverse event; BLV, bulevirtide; CP, Child‐Pugh; HCC, hepatocellular carcinoma; HDV, hepatitis delta virus; HIV, human immunodeficiency virus; RNA, ribonucleic acid; SAE, serious adverse event.

#### 4.6.1 Decompensated Cirrhosis

While the use of BLV in patients with decompensated cirrhosis is currently off‐label [42], EASL HBV guidelines recommend BLV in patients with decompensated liver disease depending on patients' risk benefit assessments [16]. BLV's potential effectiveness and safety in decompensated patients is supported in real‐world cohort studies across Europe [55, 62], with some studies also identifying improvements in Child‐Pugh cirrhosis stage with treatment [83, 84]. In a study of patients on the liver transplantation list (including decompensated patients), 15% experienced significant liver function improvement with BLV and were subsequently delisted [84], indicating BLV's potential to transform disease management prior to transplantation in patients with decompensated cirrhosis. In our experience, the objective of BLV treatment for decompensated patients is often prevention of liver disease progression. As decompensating events could make patients ineligible for transplantation, initiating BLV pre‐transplantation in the transplantation‐listed population may play a key role in maintaining transplantation eligibility.

#### 4.6.2 Hepatocellular Carcinoma

Clinicians may discuss both BLV and HCC‐specific treatments with eligible patients with HDV infection and HCC, highlighting that potential improvement in liver function with BLV can facilitate tolerability of HCC‐specific treatments. Additionally, hepatologists may be a useful resource for the other specialists involved in these patients' care (e.g., by providing recommendations to surgeons on protecting the liver while awaiting transplantation). While real‐world data on BLV are limited in this population, among 8 French patients with HDV infection and HCC on the liver transplantation list, BLV treatment led to HCC downstaging in 1 patient and 5 patients received loco‐regional therapy based on improved biochemical response, ultimately reducing liver transplantation waitlist drop‐out rates [84]. In SAVE‐D, 6% of patients had active HCC at the time of treatment initiation; no concerns related to BLV response were observed [54].

#### 4.6.3 Transplantation

Liver transplantation represents an important treatment option in patients with CHD and decompensated cirrhosis and/or HCC [15, 16], and may be needed in addition to other strategies (e.g., BLV) in eligible patients. Post‐transplantation, HBV and HDV recurrence prevention are achieved with hepatitis B immune globulin and NAs [15, 16]. The management of recurrent HDV infection post‐transplantation continues to evolve. For example, in 2024, a post‐kidney transplant patient with HDV infection achieved virologic and biochemical response and undetectable HDV RNA with BLV treatment, supporting BLV's effectiveness in solid‐organ transplantation recipients [85]. Clinicians should discuss and evaluate the possible benefit of BLV with patients on an individual basis.

#### 4.6.4 HIV/HDV Co‐Infection

The prevalence of HDV is high in patients with HBV/human immunodeficiency virus (HIV) co‐infection, particularly among individuals who inject drugs, and patients with HDV/HIV co‐infection have higher rates of HCC and liver‐related mortality than HDV‐negative patients, emphasizing the importance of connecting these patients to care [88]. In SAVE‐D and an Italian cohort study, patients with HDV/HIV co‐infection treated with BLV monotherapy had comparable response to those without HIV [54, 87]. Additionally, 58% of patients with HDV/HIV co‐infection in BuleDelta achieved virologic response with BLV treatment (with or without Peg‐IFNα) [86]. However, larger studies and additional clinical experience with BLV in this population are needed to confirm the generalizability of these results.

#### 4.6.5 Pregnancy

Interferon (IFN) regimens are contraindicated in pregnant women [89], and BLV is not recommended for use in pregnancy as human data are limited and BLV's impact on breastfeeding or fertility is unknown [42]. Doctors should clearly discuss these data limitations and reliable contraception with patients of child‐bearing potential.

For women planning pregnancy, these recommendations may be difficult to reconcile with the prospect of long‐term BLV treatment. Clinicians may have an individualized pre‐conception discussion with these patients that factors in the stage of liver disease to weigh the timing of pregnancy against BLV initiation or continuation (including safety considerations of temporarily discontinuing BLV, which may be challenging in patients with cirrhosis) and the benefits and risks of treatment. Conversations around pregnancy‐related risks associated with chronic liver disease are also critical, and shared‐decision making is important to align treatment timing with liver‐related prognosis and reproductive plans. If pregnancy occurs while a patient is on BLV, close monitoring and coordination across care teams are important to balance maternal liver disease severity against the uncertainty of fetal safety.

## 5 Future Perspectives

### 5.1 Special Populations

Data on BLV treatment are lacking in several special populations. While simulated analyses have investigated BLV dosage in paediatric patients [90], and BLV is approved for paediatric patients aged 3–18 (recommended dose depending on body weight), its safety and effectiveness has not been established in a real‐world setting [42]. Similarly, elderly patients > 65 years have been included in only some real‐world BLV studies (e.g., D‐SHIELD) [91], and the optimal treatment for patients with HBV/HDV/hepatitis C virus (HCV) triple‐infection, who often present with more severe disease than patients with HBV/HDV co‐infection, has also not been established [92]. While our clinical experience with treating elderly and HBV/HDV/HCV triple‐infected patients with BLV has not raised any concerns, this experience is limited.

### 5.2 Treatment Approaches: BLV + Peg‐IFNα Combination Therapy

EASL HDV guidelines indicate that BLV and Peg‐IFNα combination therapy may be considered in patients without Peg‐IFNα intolerance or contraindications [15]. Combination therapy was found to be effective and well‐tolerated in the MYR203 and MYR204 Phase 2/2b trials [77, 93], but has otherwise only been assessed in a few studies, including the Swedish SEE‐D clinical trial [94], two observational French cohorts [95], and an Austrian cohort study evaluating Peg‐IFNα as a potential add‐on strategy in BLV suboptimal responders [96]. Additional data are needed to establish the safety and effectiveness of combination therapy before further guidance can be established, as well as to understand the value of combination therapy in non‐responders and sub‐optimal responders to BLV monotherapy.

### 5.3 Treatment Approaches: Finite Therapy

EASL guidelines do not recommend an optimal treatment duration for BLV [15]. Per the current EMA label, BLV treatment should be continued for as long as associated with clinical benefit, and careful consideration should be taken prior to discontinuing BLV [42]. In practice, patients commonly ask about the expected duration of therapy and express a desire to discontinue treatment after a defined period. However, this decision requires careful counselling regarding the currently limited data on treatment discontinuation strategies.

In MYR301, a small subset of patients achieved sustained undetectable HDV RNA after 144 weeks of BLV monotherapy; all new HDV RNA relapses occurred within the first 48 weeks off‐treatment (most within the first 24 weeks), and patients with relapse experienced ALT increases from end‐of‐treatment and viral loads approaching baseline levels [52]. In patients with ≥ 96 weeks of on‐treatment undetectable HDV RNA, 90% had sustained undetectability 96 weeks after stopping treatment [52], demonstrating the potential value of BLV in facilitating long‐term, off‐treatment undetectable viremia. Patients with sustained undetectability also maintained ALT normalization and stable median ALT [52]. While real‐world studies evaluating off‐treatment response to BLV remain limited, in a subgroup of patients in the BuleDelta cohort who stopped treatment (BLV monotherapy or BLV and Peg‐IFNα combination therapy; all patients were HDV RNA‐negative at treatment discontinuation), 47% of patients had sustained undetectable HDV RNA 96 weeks after stopping treatment [97]. In clinical practice, treatment discontinuation decisions should rely on long‐term, on‐treatment HDV RNA undetectability confirmed with the most sensitive assay available (and use the same assay as was used on‐treatment) as assays used in clinical trials may not be available in real‐world practice, as aforementioned, followed by close monitoring for HDV RNA relapse. In cases of relapse, real‐world data suggest BLV re‐initiation (with or without Peg‐IFNα) may lead to re‐achievement of virologic response [98, 99].

While the potential for finite therapy with BLV from clinical trials is promising, the overall rates only represent a subset of patients and further research is required if this is to be a realistic goal for the majority of patients. Doctors should clearly inform patients of the importance of not discontinuing treatment without medical consultation.

### 5.4 Future Therapeutic Goals

Ultimately, there exists an unmet need for a curative therapy for HDV infection [100]. A functional HBV cure will be central to eliminating HDV infection, and future therapeutic strategies may target both HBV and HDV [100]. While undetectable HDV RNA is a key endpoint of many treatments, sustained HBsAg loss is also clinically relevant given the role of HBsAg in HDV dissemination [100]. As BLV does not act on the HBV genome, BLV has not been found to impact HBsAg when administered as monotherapy [100].

Novel treatment options spanning a variety of MoAs are in development and under evaluation in clinical trials [101]. BLV's effectiveness in reducing ALT and HDV RNA levels during long‐term treatment suggests that BLV may remain a key component of HDV infection management even as the therapeutic landscape evolves [50, 51, 52]; BLV may be increasingly used in combination with emerging agents with complementary MoAs to achieve functional HBV/HDV cure and treat non‐responders to BLV monotherapy. Ongoing research and drug development also raise the possibility of more potent antiviral strategies for HDV infection and, ultimately, finite treatment approaches aimed at achieving sustained off‐treatment responses.

## 6 Conclusion

There is substantial heterogeneity in HDV epidemiology across different European regions, and unmet needs remain in patient identification, diagnosis, and linkage‐to‐care. Increased awareness among doctors of the CHD disease course and universal screening guidelines represents a key step in facilitating patient finding and initiating the care cascade. Reflex testing strategies are critical to identifying undiagnosed patients early in the disease course, particularly in vulnerable populations such as migrants. The approval of BLV 2 mg monotherapy for the treatment of CHD in Europe marks a significant advance in CHD management; BLV is the first CHD treatment option with a large eligible patient population, and its availability has led to an increased urgency to connect patients to care, reflected in increased screening efforts and the accumulation of real‐world evidence and relevant learnings surrounding best practices for CHD management. Discussions with patients around disease management have become increasingly proactive and optimistic with an approved treatment option available; during these conversations, it is critical for physicians to set evidence‐based treatment expectations and goals and to provide comprehensive guidance and ongoing support to patients. Clinical trial and real‐world data consistently indicate that long‐term BLV monotherapy is safe and effective, even in patients with cirrhosis, and may improve liver function tests and liver stiffness measurements, which are important motivators for patients. Treatment adherence and persistence are generally high, and supportive, patient‐centred approaches to care are important for successful disease management. Future research is needed to establish the safety and effectiveness of BLV in certain special populations and determine the optimal duration of treatment.

## Author Contributions

Substantial contributions to study conception and design: M.R., M.P., H.K.K., S.L., E.D., S.I., L.G., K.S., F.A.C., P.L., S.A., and H.W.; substantial contributions to analysis and interpretation of the data: M.R., M.P., H.K.K., S.L., E.D., S.I., L.G., K.S., F.A.C., P.L., S.A., and H.W.; drafting the article or revising it critically for important intellectual content: M.R., M.P., H.K.K., S.L., E.D., S.I., L.G., K.S., F.A.C., P.L., S.A., and H.W.; final approval of the version of the article to be published: M.R., M.P., H.K.K., S.L., E.D., S.I., L.G., K.S., F.A.C., P.L., S.A., and H.W.

## Funding

Support for third‐party writing assistance for this article, provided by Lauren Konieczynski and Océane Parker, Costello Medical, US, was funded by Gilead Sciences, Inc. in accordance with Good Publication Practice (GPP 2022) guidelines (https://www.ismpp.org/gpp‐2022).

## Ethics Statement

The authors have nothing to report.

## Consent

The authors have nothing to report.

## Conflicts of Interest

Monica Radu has received grants or contracts from Gilead Sciences Inc.; has received payment or honoraria from Gilead Sciences Inc.; has received support for attending meetings and/or travel from Gilead Sciences Inc.; has participated in advisory boards for Gilead Sciences Inc. Margarita Papatheodoridi, Habiba Kamal Khodir have nothing to disclose. Sabela Lens has received grants or contracts from Gilead Sciences Inc.; has received consulting fees from Gilead Sciences Inc., GSK, AbbVie, and Roche; has received payment or honoraria from Gilead Sciences Inc., GSK, AbbVie, and Roche; has received support for attending meetings and/or travel from Gilead Sciences Inc. and AbbVie; is a member of the EASL scientific committee. Elisabetta Degasperi has received grants or contracts from Gilead Sciences Inc.; has received consulting fees from Gilead Sciences Inc., Ipsen, Mirum, and Roche; has received payment or honoraria from Gilead Sciences Inc., Ipsen, and Mirum; has received support for attending meetings and/or travel from Gilead Sciences Inc.; has participated in advisory boards for Gilead Sciences Inc. and Mirum. Speranta Iacob: has received support for attending meetings and/or travel from Gilead Sciences Inc. and AbbVie. Liliana Gheorghe: has received grants or contracts from Gilead Sciences Inc.; has received consulting fees from Gilead Sciences Inc., AbbVie, Lilly, Novo Nordisk, Innergy, and Ewopharma; has received payment or honoraria from Gilead Sciences Inc., AbbVie, Lilly, Novo Nordisk, Innergy, Takeda, and Sanofi; has received support for attending meetings and/or travel from Gilead Sciences Inc., AbbVie, and Takeda; has participated in advisory boards for Eli Lilly, Novo Nordisk, AbbVie. Katja Steppich: nothing to disclose. Florin Alexandru Caruntu: has received grants or contracts from Gilead Sciences Inc.; has received payment or honoraria from Gilead Sciences Inc.; has received support for attending meetings and/or travel from Gilead Sciences Inc.; has participated in advisory boards for Gilead Sciences Inc. Pietro Lampertico: has received advisor and speaker bureau fees from Gilead Sciences Inc., Roche Pharma/Diagnostics, GSK, AbbVie, Janssen, Myr Pharma, EIGER, Antios, Aligos, Vir, Grifols, Altona, Roboscreen; has received consulting fees and grants from Gilead Sciences Inc., and AbbVie; has consulted for Gilead Sciences Inc., AbbVie, Vir Biotechnology Inc., Arrowhead, Transgene, BMS, ML. Soo Aleman: has received grant funding from Gilead Sciences Inc.; has received honoraria for lectures from Gilead Sciences Inc.; has participated in advisory boards for Gilead Sciences Inc. and Ribocure. Heiner Wedemeyer has received grants or contracts from Abbott Laboratories & Abbott Molecular Inc. and Biotest AG; has received consulting fees from and participated in a data safety monitoring or advisory board for Abbott Laboratories & Abbott Molecular Inc., Aligos Therapeutics Inc., Bluejay Therapeutics, Bristol‐Myers‐Squibb, F. Hoffmann‐La Roche Ltd., Gilead Sciences Inc., GlaxoSmithKline Services Unlimited, Ribocure Pharmaceuticals AB, Vir Biotechnology Inc.; has received payment or honoraria from Biotest AG and Gilead Sciences Inc.

## Data Availability

The authors have nothing to report.
